# Determinants of Face Mask Utilization to Prevent Covid-19 Pandemic among Quarantined Adults in Tigrai Region, Northern Ethiopia, 2020

**DOI:** 10.1177/10547738211013219

**Published:** 2021-05-06

**Authors:** Mekonnen Haftom, Pammla M. Petrucka

**Affiliations:** 1 Adigrat University, Ethiopia; 2 University of Saskatchewan, Regina, Canada

**Keywords:** determinants, face masks, Covid-19 pandemic, personal protection equipment, pandemics, adult, Ethiopia

## Abstract

A face mask is a vital component of personal protective equipment to prevent potentially contagious respiratory infections. There was a lack of evidence showing the proportion and determinants of face mask use in Ethiopia. Therefore, this study aimed to identify face mask utilization determinants to prevent spread of the Covid-19 pandemic among quarantined adults in Tigrai region, northern Ethiopia. A total of 331 participants selected using a systematic random sampling method were included in the study. An interviewer-administered questionnaire was employed. After describing the variables using frequencies, means, and standard deviations, multivariable logistic regression determined factors associated with face mask utilization to prevent COVID-19 spread. The study participants were primarily males (70%) and mean age was 30.5 (*SD* = 11) years. Nearly half of the participants reported they did not wear a face mask when leaving home. Face mask utilization was significantly associated with knowledge score, employment status, gender, age, and educational status of the study participants.

## Introduction

Corona virus disease—2019 (Covid-19) was declared a public health emergency of international concern in January 2020 (Cucinotta & Vanelli, 2020; Gagliano et al., 2020). As of May 27, 2020, the disease has caused 5,488,825 cases and 349,095 deaths worldwide. In Africa, 85,815 cases and 2,308 deaths have been reported (World Health Organization [WHO], 2020a). This disease affects all ages, but its severity was higher among the aged and those with underlying chronic diseases (Davies et al., 2020). For instance, the death rate among older people was 48% compared to younger people (Li et al., 2020). The pandemic caused rapid loss of life, joblessness, and deterioration of the healthcare delivery systems, national, and global economies (Lzvorski et al., 2020). In September 2019, it was projected that there would be a catastrophic respiratory disease that is rapidly moving and could result in the deaths of between 50 and 80 million people while impacting the world economy by 5% (Broberg, 2020). In Ethiopia, the first case of Covid-19 was confirmed on March 13, 2020, in Addis Ababa.

The pandemic has since spread to all parts of the country. As of May 27, 2020, the Africa news reported 731confirmed cases and six deaths (Addis News, 2020). The use of a face mask is one of the critical measures to prevent the virus’ spread. However, it should be integrated with physical distancing, hand hygiene, and related preventive measures (WHO, 2020b, 2020c, 2020d). Infected individuals can be asymptomatic or may present with mild to severe manifestation. The risk of transmission from asymptomatic individuals is high, making the disease difficult to control (Howard et al., 2020; Wang et al., 2020; WHO, 2020e). Anticipating the disease’s pandemic spread continues in developing countries, its impact will be more severe than that seen in the world’s developed regions due to weak or unresponsive health systems (Moorthy et al., 2020).

A face mask is a vital component of personal protective equipment to prevent potentially contagious respiratory infections (Wei et al., 2020). There were controversies in using a mask. Issues regarding its effectiveness and fear of shortage of resources were some of the claimed reasons some experts recommended not to use a face mask (Madad et al., 2020). However, pieces of evidence also showed that mask use could decrease disease transmission and mortality (Chu et al., 2020; Eikenberry et al., 2020; Feng et al., 2020; Klompas et al., 2020; WHO, 2020b). No research studies have been identified to determine the proportions and determinants of face mask utilization to prevent Covid-19 in Ethiopia. Therefore, this study aimed to identify face mask utilization determinants to prevent the Covid-19 pandemic among quarantined adults in the Tigrai region, northern Ethiopia.

## Methods and Materials: Study Design, Setting, and Population

This manuscript is a part of a broader study considering knowledge, attitude, and practice towards the Covid-19 pandemic in Tigrai region. An institutional-based cross-sectional study was conducted among adults quarantined in Tigrai Region, Northern Ethiopia. The criteria for quarantine were international travel history, travel history from Addis Ababa (where community transmission started), and exposure to positive individuals.

A total of 331 randomly selected participants aged 18 to 69 were interviewed from May 15 to 27, 2020. Using a single population proportion formula to calculate sample size assuming a 95% confidence level, 5% margin of error, and 50% of the population wear a face mask when leaving home (*p* = .5), it yielded a calculated sample size of *n* = 384. Since the total source population was less than 10,000 (*N* = 2102), the estimated sample size (*n* = 384) was reconsidered using a correction formula. The final sample size after correction (nf = 326) with the addition of a 5% non-response rate yielding a sample size of 343.

## Recruitment Procedure

Multi-stage sampling was used. The region is clustered into zones (*n* = 7). Three quarantine centers in each zone were randomly selected. The total sample size is proportionally allocated to the number of confined and isolated individuals over the most recent 2 weeks. The sampling interval was determined by dividing the number of individuals admitted to the centers in the selected 2 weeks by the sample allocated to that center, which yield a *k* of 7. A systematic random sampling method was used to choose the study participants. The first participant (between 1 and 7) was selected using a lottery method. Therefore, every seventh individual was enrolled in the study until the calculated sample size was achieved.

## Study Instrument and Data Collection Procedure

The instrument is adapted from similar studies done in Tanzania and China. The questionnaire was found to be valid, reliable, and used in other studies (Advani et al., 2020; Sommerstein et al., 2020). The tool had three sections: the socio-demographic profile (sex, age, educational status, and employment status); knowledge about clinical characteristics of COVID-19 (items 1–4); knowledge about modes of transmission of COVID-19 (items 5–8); and knowledge about prevention and control of COVID-19 (items 9–13). Each item has “true” (coded as 0), “false” (coded 1), and “I don’t know” (coded 2) options. For analysis, it was again re-coded to sum the total scores as 1 for correct answers and 0 for both incorrect and I don’t know responses. The possible score ranges from 0 to 13; with the higher scores indicating good knowledge. For the purposes of this study, a score ≥10 being acceptable levels of knowledge (Zhong et al., 2020). The third section was about face mask utilization when leaving home. We collected data using a self-administered questionnaire for the literate participants. If the respondent was illiterate, the data were collected using face-to-face interviews by trained data collectors.

As part of the quarantine protocol, only one adult was allowed to be in a single room. Therefore, by default privacy was kept by keeping distance between the interviewer and the interviewee. Interviews were based on convenience for the individual participant.

## Data Quality Assurance and Control

The questionnaire was prepared in English and back-translated to the local language Tigrigna. Also, the tool’s accuracy was checked by back translating to English by experts who were blind to the original instrument.

Before starting the data collection, a pre-test was done on 10% (34 individuals) of the total sample size, and an amendment was made accordingly. Also, data were collected by trained data collectors, and the completed questionnaires’ completeness was ensured thorough manual checking daily. Data collectors were trained for 3 days regarding the study’s objectives, data collection procedure, and ethical considerations a week ahead of the actual data collection.

## Data Processing and Analysis

Data were entered and cleaned using Epi-data manager™ version 3.1, and Statistical Package for the Social Sciences (SPSS)™ version was used for analysis. Frequency, means, and standard deviations (SDs) of participants’ demographic characteristics were calculated to identify possible baseline differences and presented using tables and text. The binary logistic regression model was used to determine the magnitude, direction, and strength of association between demographic profiles, knowledge scores, and face mask utilization. Variables significant at *p* < .25 with a face mask utilization were selected for a multivariate logistical regression model analysis. An odds ratio with 95% confidence level was computed, and *p*-value <.05 was considered a significant association.

## Ethical Consideration

The study considered the Helsinki Declaration. The Tigrai Regional Health Bureau (TRHB) approved the study. A support letter was submitted to the region’s selected quarantine centers, and letters of permission were secured from each center’s administrative bodies and coordinators. Participants recruited to the study received a verbal explanation of the research and were provided a written information sheet. They were informed regarding compensation (no compensation was provided). All potential participants who agreed to participate provided written consent to continue with the interviews. The confidentiality of information obtained was kept, and respondents’ names were not recorded.

## Results

### Demographic Characteristics

Of the 343 approached participants, 331 completed the questionnaire, making the response rate 96.5%. Table 1 shows the socio-demographic profiles and knowledge scores of the study participants. The mean age was 30.5 years (*SD* = 11). Seventy percent of the participants were males, and more than half of the study participants (59.8%) were in the age range 18 to 29. About one-third of the study participants were self-employed (31.1%) and have completed secondary school (36%). The mean (standard deviation) knowledge score was 8.73 (±2.64). The rate of the correct answer was 67.2% (8.73/13 × 100). Less than half of participants [142 (42.9%) (95% confidence interval (CI): 37.5%–48%)] were knowledgeable (scored ≥10). The remaining 189 (57.1%) (95% CI: 52%–62.5%) were not knowledgeable. Forty-six percent (95% CI: 40.2%–51%) of the participants did not use a face mask when leaving home.

**Table 1.** Demographic Profiles of Study Participants (*n* = 331).

Variables	Category	*n*	%
Gender	Male	230	69.5
	Female	101	30.5
Age	18–29	198	59.8
	30–49	104	31.4
	Above 50	29	8.8
Occupation	Student	39	11.8
	Public sector	28	8.5
	Private sector	24	7.3
	Self-employed	103	31.1
	Not employed	80	24.2
	Farmer	38	11.5
	Retired	4	1.2
	Others	15	4.5
Educational status	Illiterate	37	11.2
	Read and write	21	6.3
	Primary school	90	27.2
	Secondary school	119	36
	Illiterate	37	11.2
Knowledge score	Acceptable	142	42.9
	Not acceptable	189	57.1

### Determinants of Low Utilization of Face Mask

Multivariable logistic regression showed that males were 2 (95% CI: 1.1–3.2) times more likely to wear masks when leaving home. The odds of mask utilization amongst the age group of ≥50 years and 30 to 49 years were 12 (95% CI: 3.2–45.1) and 2.5 (95% CI: 1.4–4.6) times higher than those aged 18 to 29. Controlling the effect of being a student, the odds of mask use among public and private sector employees were 5.2 (95% CI: 1.2–23) and 3.6 (95% CI: 1.1–12.5). The odds of mask use among farmers were 68% [0.32(95% CI: 0.1–0.9)] lower than students. Those who completed college or university were 8.1 (95% CI: 2.1–31.7) times more likely to use a mask when leaving home than those who cannot read and write. The odds of mask use amongst knowledgeable participants were 2 (95% CI: 1.2–3.3) times higher than the non-knowledgeable counterparts (Table 2).

**Table 2.** Multivariable Logistic Regression on Socio-demographic Characteristics, Knowledge Score, and Utilization Face Mask (*n* = 331).

Variables	Category	Face mask used (%)	Face mask not used (%)	COR (95% CI)	AOR (95% CI)
Gender	Male	132 (39.9)	98 (29.6)	2.7 (1.7–4.3)	2 (1.1–3.2)**
	Female	48 (14.5)	53 (16)	Ref	Ref
Age	18–29	110 (33.2)	88 (26.6)	Ref	Ref
	30–49	57 (17.2)	47 (14.2)	2 (1.2–3.2)	2.5 (1.4–4.6)***
	Above 50	13 (3.9)	16 (4.8)	2.9 (1.2–6.9)	12 (3.2–45.1)***
Occupation	Student	22 (6.6)	17 (5.1)	Ref	Ref
	Public sector	19 (5.7)	9 (2.7)	6.9 (1.8–26.9)	5.2 (1.2–23)**
	Private sector	11 (3.3)	13 (3.9)	3.6 (1.1–11.5)	3.6 (1.1–12.5)**
	Self-employed	68 (20.5)	35 (10.6)	1.7 (0.8–3.5)	1.7 (0.8–3.9)
	Not employed	39 (11.8)	41 (12.4)	0.7 (0.3–1.4)	0.9 (0.4–2)
	Farmer	15 (4.5)	23 (6.9)	0.3 (0.1–0.7)	0.32 (0.1–0.9)**
	Retired	3 (0.9)	1 (0.3)	2.7 (0.3–28)	1.7 (0.1–19.5)
	Others#	3 (0.9)	12 (3.6)	0.2 (0.1–0.9)	0.4 (0.1–2)
Educational status	Illiterate	11 (3.3)	26 (7.9)	Ref	Ref
	Read and write	8 (2.4)	13 (3.9)	3.3 (1–10.6)	4.9 (1.2–20.5)**
	Primary school	53 (16)	37 (11.2)	5.4 (2.1–13.5)	6.3 (1.7–22.6)***
	Secondary school	71 (21.5)	48 (14.5)	5.9 (2.4–14.5)	6.9 (2–24.4)***
	College or university	37 (11.2)	27 (8.2)	10.5 (4–28.2)	8.1 (2.1–31.7)***
Knowledge score	Acceptable	94 (24.4)	48 (14.5)	2.8 (1.7–4.3)	2 (1.2–3.3)*
	Not acceptable	86 (26)	103 (31)	Ref	Ref

*Note.* AOR = adjusted odds ratio; COR = crude odds ratio;

#Others = driver, housewife, daily laborers.

* p < .05. **p < .01. ***p < .001.

## Discussion

After the virus emerged in China, it is argued that information was suppressed and erroneous. China initially reported the novel virus showed no evidence of human-to-human transmission (Azlan et al., 2020; Byanaku & Ibrahim, 2020). As per the authors’ knowledge, this is the first study of persons in quarantine centers to assess mask utilization determinants to prevent Covid-19 in Ethiopia. This study has provided evidence-based data regarding face mask utilization. Nearly half of the study participants have used face masks when leaving home in recent days.

The lack of use of face masks is not consistent with the recommendations (Davidson, 2020; Greenhalgh et al., 2020; Ogden, 2020) that everyone should wear a mask when going out. Some countries (Mahase, 2020) have declared state mandates on mask use when leaving home, while using face mask was underemphasized in other countries (Greenhalgh et al., 2020). However, Ethiopia did not declare mandates on face mask use until the time of data collection. This position could be due to controversies to use or not to use face masks (Lyu & Wehby, 2020; Tso & Cowling, 2020; Wang et al., 2020) and may have contributed to the low report of mask used in our study area.

Our findings indicated that educational status, knowledge score, age, gender, and occupation of study participants were significant predictors of mask use. The results of this study demonstrate that a higher level of education and acceptable level of knowledge towards COVID-19 (Advani et al., 2020; Chen et al., 2020; Fisman et al., 2020; Kuo et al., 2011; Taylor et al., 2009; Wong et al., 2020) increases face mask use by participants when leaving home. These findings indicate younger participants were less likely to use a face mask, which aligns with other studies (Chen et al., 2020; Lau et al., 2007) that older people tended to use a mask. COVID-19 is less common amongst youngsters (Bai et al., 2020; Lee et al., 2020; Tang & Wong, 2004; Tomar et al., 2020) compared to their older adult counterparts, which may contribute to the low utilization of face masks among those aged 18 to 29 compared to their elder counterparts.

Some studies (Brodin, 2020; Lee et al., 2020) were inconsistent with our finding that females were more likely to use face masks. Participants employed in private and public sectors are more likely to use face masks than students. However, farmers were least likely to use a face mask. The reason for this variability could be explained as employed participants might be obliged to use masks when they are on duty, and limited access to face masks and costs might cause farmers not to use them.

## Limitation

This is a small-scale cross-sectional study, which may limit the generalizability of the results across different periods and different communities. Therefore, a large-scale country level study would have provided more generalizable data. When the rapid response team traced the study participants, they provided education regarding the pandemic on the way to the quarantine centers before the entry interview (data collection), which might have made study participants more aware of mask use compared to the community as a whole. Social desirability bias was likely regarding face mask use.

## Conclusion

Nearly half of the total participants did not use a face mask when leaving home. Employment status level of education, gender, age, and knowledge score were significantly associated with face mask utilization by adults in Tigrai Region, Northern Ethiopia.
